# Correction: Noguchi et al. PCR-Based Screening of Spinal Muscular Atrophy for Newborn Infants in Hyogo Prefecture, Japan. *Genes* 2022, *13*, 2110

**DOI:** 10.3390/genes14030759

**Published:** 2023-03-21

**Authors:** Yoriko Noguchi, Ryosuke Bo, Hisahide Nishio, Hisayuki Matsumoto, Keiji Matsui, Yoshihiko Yano, Masami Sugawara, Go Ueda, Yogik Onky Silvana Wijaya, Emma Tabe Eko Niba, Masakazu Shinohara, Yoshihiro Bouike, Atsuko Takeuchi, Kentaro Okamoto, Toshio Saito, Hideki Shimomura, Tomoko Lee, Yasuhiro Takeshima, Kazumoto Iijima, Kandai Nozu, Hiroyuki Awano

**Affiliations:** 1 Department of Clinical Laboratory, Kobe University Hospital, 7-5-1 Kusunoki-cho, Chuo-ku, Kobe 650-0017, Japan; 2 Department of Pediatrics, Kobe University Graduate School of Medicine, 7-5-1 Kusunoki-cho, Chuo-ku, Kobe 650-0017, Japan; 3 Department of Community Medicine and Social Healthcare Science, Kobe University Graduate School of Medicine, 7-5-1 Kusunoki-cho, Chuo-ku, Kobe 650-0017, Japan; 4 Department of Occupational Therapy, Faculty of Rehabilitation, Kobe Gakuin University, 518 Arise, Ikawadani-cho, Nishi-ku, Kobe 651-2180, Japan; 5 Department of Pediatrics, Kobe City Medical Center General Hospital, 2-1-1 Minatojimaminami-machi, Chuo-ku, Kobe 650-0047, Japan; 6 Department of Pediatrics, Ueda Hospital, 1-1-4 Kunikadori, Chuo-ku, Kobe 651-0066, Japan; 7 Department of Biochemistry, Faculty of Medicine, Public Health, and Nursing, Universitas Gadjah Mada, Jalan Farmako, Yogyakarta 55281, Indonesia; 8 Laboratory of Molecular and Biochemical Research, Biomedical Research Core Facilities, Juntendo University Graduate School of Medicine, Hongo, Bunkyo-ku, Tokyo 113-8421, Japan; 9 Faculty of Nutrition, Kobe Gakuin University, 518 Arise, Ikawadani-cho, Nishi-ku, Kobe 651-2180, Japan; 10 Instrumental Analysis Center, Kobe Pharmaceutical University, 4-19-1 Motoyamakitamachi, Higashinada-ku, Kobe 658-8558, Japan; 11 Department of Pediatrics, Ehime Prefectural Imabari Hospital, 4-5-5 Ishi-cho, Imabari 794-0006, Japan; 12 Department of Neurology, National Hospital Organization Osaka Toneyama Medical Center, 5-1-1 Toneyama, Toyonaka 560-8552, Japan; 13 Department of Pediatrics, Hyogo Medical University, 1-1 Mukogawacho, Nishinomiya 663-8501, Japan; 14 Hyogo Prefectural Kobe Children’s Hospital, 1-6-7 Minatojimaminami-machi, Chuo-ku, Kobe 650-0047, Japan

## Error in References Section

The authors wish to make the following correction to this paper [1]. There were some errors about reference citations in the original publication. Firstly, there was a wide range of mismatched pairings of contents in the text and cited references in the original publication. This was due to the skipping of one reference in the Reference section, which led to the shift of the reference number. Secondly, two articles cited in the original version were not closely related to the text. Thirdly, a mis-citation of one paper occurred in the original version.

Therefore, one reference (Arnold et al., 2015) has been added, two articles have been replaced by two other articles (i.e., Kraszewski et al., 2018 and Boemer et al., 2021), and a number of mis-cited references in the text have been deleted. With these corrections, the order of some references has been adjusted, and the numbers of cited references in the text have been changed accordingly.

All corrections to the text are shown below. The authors state that the scientific conclusions are unaffected. These corrections were approved by the academic editor. The original publication has also been updated.

1. A new reference: “Arnold, W.D.; Kassar, D.; Kissel, J.T. Spinal muscular atrophy: Diagnosis and management in a new therapeutic era. *Muscle. Nerve*. **2015**, *51*, 157–167.” has been added at the position after Reference 2.
2. Reference 23 (Reference 24 after re-numbering): “Kariyawasam, D.S.T.; D’Silva, A.; Lin, C.; Ryan, M.M.; Farrar, M.A. Biomarkers and the Development of a Personalized Medicine Approach in Spinal Muscular Atrophy. *Front. Neurol.* **2019**, *10*, 898.” has been replaced by “Kraszewski, J.N.; Kay, D.M.; Stevens, C.F.; Koval, C.; Haser, B.; Ortiz, V.; Albertorio, A.; Cohen, L.L.; Jain, R.; Andrew, S.P.; et al. Pilot study of population-based newborn screening for spinal muscular atrophy in New York state. *Genet. Med.* **2018**, *20*, 608–613.”
3. Reference 24 (Reference 25 after re-numbering): “Boemer, F.; Caberg, J.-H.; Dideberg, V.; Dardenne, D.; Bours, V.; Hiligsmann, M.; Dangouloff, T.; Servais, L. Newborn Screening for SMA in Southern Belgium. *Neuromuscul. Disord.* **2019**, *29*, 343–349.” has been replaced by “Boemer, F.; Caberg, J.H.; Beckers, P.; Dideberg, V.; di Fiore, S.; Bours, V.; Marie, S.; Dewulf, J.; Marcelis, L.; Deconinck, N.; et al. Three years pilot of spinal muscular atrophy newborn screening turned into official program in Southern Belgium. *Sci. Rep*. **2021**, *11*, 19922.”
4. In the first paragraph of section ***2.1. Patients and Fetuses with SMA***, the number of mis-cited reference (Reference 1) has been deleted.
5. Due to the addition of the new reference and citation errors of references, the re-numbering of the citation in the text has been done.

(1) In the first paragraph of section ***4.2. Incidence Rates of SMA Based on Newborn Screening***, refs. [23–28] has changed to [23–29] (after renumbering).
(2) In the second paragraph of section ***4.2. Incidence Rates of SMA Based on Newborn Screening***, ref. [29] has changed to [30] (after renumbering).
(3) In the first paragraph of section ***4.3. False-Positive Cases in Hyogo Prefecture***, refs. [19,26,30–33] has changed to [19,26,31–33] (after renumbering), refs. [23,34,35] to [23,35,36] (after renumbering), ref. [34] to [35,36] (after renumbering), and ref. [35] to [36] (after renumbering).
(4) In the third paragraph of section ***4.3. False-Positive Cases in Hyogo Prefecture***, ref. [36] has changed to [37] (after renumbering).
(5) In the first paragraph of section ***4.4. SMA-NBS and Pre-Symptomatic Treatment***, refs. [17,37,38] has changed to [17,38,39] (after renumbering).
(6) In the second paragraph of section ***4.4. SMA-NBS and Pre-Symptomatic Treatment***, ref. [39] has changed to [40] (after renumbering).
(7) In the fourth paragraph of section ***4.4. SMA-NBS and Pre-Symptomatic Treatment***, ref. [40] has changed to [41] (after renumbering).
(8) In the second paragraph of section ***4.5. SMA-NBS and Post-Symptomatic Treatment***, ref. [41] has changed to [42] (after renumbering), ref. [42] to [43] (after renumbering), ref. [43] to [44] (after renumbering), and ref. [44] to [45] (after renumbering).
(9) In the third paragraph of section ***4.5. SMA-NBS and Pre-Symptomatic Treatment***, each ref. [45] has changed to [46] (after renumbering).
